# An EEG Dataset of Neural Signatures in a Competitive Two-Player Game Encouraging Deceptive Behavior

**DOI:** 10.1038/s41597-024-03234-y

**Published:** 2024-04-16

**Authors:** Yiyu Chen, Siamac Fazli, Christian Wallraven

**Affiliations:** 1 Department of Artificial Intelligence, Korea University, Seoul, 02841 South Korea; 2 Department of Computer Science, Nazarbayev University, Astana, 010000 Kazakhstan; 3 Department of Brain and Cognitive Engineering, Korea University, Seoul, 02841 South Korea

**Keywords:** Decision, Cooperation

## Abstract

Studying deception is vital for understanding decision-making and social dynamics. Recent EEG research has deepened insights into the brain mechanisms behind deception. Standard methods in this field often rely on memory, are vulnerable to countermeasures, yield false positives, and lack real-world relevance. Here, we present a comprehensive dataset from an EEG-monitored competitive, two-player card game designed to elicit authentic deception behavior. Our extensive dataset contains EEG data from 12 pairs (N = 24 participants with role switching), controlled for age, gender, and risk-taking, with detailed labels and annotations. The dataset combines standard event-related potential and microstate analyses with state-of-the-art decoding approaches of four scenarios: spontaneous/instructed truth-telling and lying. This demonstrates game-based methods’ efficacy in studying deception and sets a benchmark for future research. Overall, our dataset represents a unique resource with applications in cognitive neuroscience and related fields for studying deception, competitive behavior, decision-making, inter-brain synchrony, and benchmarking of decoding frameworks in a difficult, high-level cognitive task.

## Background & Summary

Deceiving another person is an intricate and multifaceted human behavior, the neurological basis of which has become an intense focus of research. Understanding the underlying neural mechanisms of deception (or lying) is essential for advancements in fields such as law, psychology, and clinical domains. Modern neuroimaging techniques, notably functional magnetic resonance imaging (fMRI) and electroencephalography (EEG), have played pivotal roles in advancing our understanding of the neural underpinnings of such deceptive behaviors. These technologies have superseded traditional polygraph approaches, offering deeper insights into neural processes. For example, the concealed information test (CIT) has been widely explored using EEG to detect specific neural responses during deception^1–5^.

While CIT-based lie detection has provided valuable insights into the neural mechanisms of deception, it is important to consider its inherent limitations. Importantly, its accuracy can be undermined by participants’ use of countermeasures, such as focusing on irrelevant stimuli^6,7^. Additionally, the CIT’s reliance on memory recognition^8^ rather than actual deceit may produce false positives among innocent individuals exposed to crime details^9,10^. Furthermore, the controlled settings of CIT experiments often lack the practical motivations present in real-world deception, potentially affecting their ecological validity. There is a pressing need to bridge these gaps, specifically in ensuring that the neural signatures captured truly reflect deceptive behavior in a real-world context. Game-based designs are emerging as potential alternatives for studying lying behavior, with several studies having incorporated such designs to assess spontaneous lying and truth-telling actions^11–15^.

In view of the growing significance of these research areas and the need for comprehensive datasets, this paper provides a novel dataset related to deceptive behavior, obtained from a competitive, two-person-based card game task employing EEG. The two-player deception game task is designed to induce real-world deceptive behavior, thereby augmenting the depth and breadth of existing deception and lie detection research. By combining spontaneous and instructed lying/truthful behaviors, this dataset offers a rich source of information for future research endeavors. Additionally, in recognizing the potential confounds related to risk-taking behavior and psychological arousal, our experimental design integrates the balloon analog risk-taking (BART) test^16,17^, thereby enhancing the granularity and robustness of our dataset. Furthermore, our task integrates interpersonal dynamics into decision-making, providing a fertile ground for insights into inter-brain interactions.

Numerous public datasets are available that span a range of tasks for studying cognitive functions. These range from simple tasks that enable cognitive processing, such as conflict control, language production, and cognitive inference, to more complex ones, such as visual / speech imagery, working memory, and decision making, as detailed in Table 1. However, datasets addressing advanced cognitive processes, especially deception, remain very few. Our dataset fills this gap, employing a game-based design to stimulate high-level cognitive deception. It is comprehensive and large scale, featuring data from 24 participants for each player role, and offers fully pre-processed, synchronized, and labeled data for approximately 121 trial epochs per condition. Notablely, to the best of our knowlage, our dataset is the first to showcasing two-player interactions that highlight advanced decision-making. Given its distinctive design, our dataset has the potential to significantly advance the understanding in the realm of cognitive deception and interpersonal decision-making.

**Table 1:** Summary of EEG Dataset papers of Higher-level Cognitive Processing.

Cognitive Function	Task	Number of Participants	Trials per Condition
Inner speech^50^	Up, down, left, right in spanish	N = 10	45–60 trials^1^
Visual & Sound Imagery^51^	Audio, orthographic, pictorial perception and imagination	N = 12	64–150 trials^2^
Working Memory^52^	N-back (0-Back, 1-Back and 2-Back)	N = 29	9 blocks
Working Memory^53^	Digit span	N = 85	108 trials
Working Memory^54^	Discriminate seen and unseen pictures	N = 20	40 trials
Conflict control^55^	Stroop task	N = 21	32 trials
Conflict control^56^	Stroop task	N = 21	32 trials
Conflict control^52^	Arrow-based Eriksen flanker task	N = 29	90 trials
Decision making^57^	Food, image semantic category, and word semantic category choice using mouse-tracking	N = 31	320 trials
Decision making^58^	Drive car back to the center of the lane using steering wheel after drift event occurs	N = 27	615–7269 trials^3^
Language production^54^	Picture naming and spelling	N = 23	148 trials
Language production^54^	Picture and auditory naming	N = 20	120 (pictrue), 80 (audio)
Cognitive inference^59^	Extended Multi-source Interference Task (MSIT+)	N = 42	102 ± 7(00), 105 ± 7(S0), 94 ± 9(F0), 98 ± 9(FS)
Mental workload^52^	MATB-II	N = 29	9 trials

Overall, our dataset will serve to provide new insights into the neuronal mechanisms of lying behavior and represents a significant addition to the field. In particular, it encompasses:

- Comprehensive EEG data collected from participants engaged in a two-person-based card game designed to induce real-world lying behavior.
- Annotations of four different experimental conditions, including spontaneous or instructed decisions, and truthful or lying behaviors.
- Demographic information and psychological assessments, including measures to control for risk-taking tendencies.
- Fully-processed data to enhance efficiency during two-player analyses, eliminating complexities associated with data synchronization. This includes pre-processing, time-stamping, synchronization, epoching, and labeling.
- Benchmark results from Event-Related-Potential (ERP) analysis, microstate analysis, and deep-neural-network decoding for the four experimental conditions.

## Methods

### Participants

24 participants (12 males and 12 females, aged 19–34, mean = 25 yrs, SD =  ± 4.34) participated in the experiment. All had normal or correct-to-normal visual acuity and none of them had a history of neurological disease or injury. The participants were naïve to the card game paradigm and gave written informed consent before the start of the experiment and received payment of around 10US$ per hour for taking part in the study. The experiment was conducted in accordance with the tenets of the Declaration of Helsinki and received IRB approval with the number KUIRB-2019-0043-01.

### Apparatus

EEG was recorded with a total of 31 electrodes at a sampling frequency of 500 Hz, using BrainAmp amplifiers and EasyCaps with an active electrode system (Brain Products, Munich, Germany). The measurements were performed with 30 EEG electrodes, namely: Fp2, F9,7,3,z,4,8,10, FC5,1,2,6, T7,8, C3,z,4, CP5,1,2,6, P7,3,z,4,8, PO3,4, O1,z,2, as well as one EOG electrode below the right eye (EOGv1). During the initial recordings, we encountered a connection issue with the Oz electrode within the recording devices. This problem compromised the reliability of data from the Oz electrode. As a result, to maintain the integrity of our study, we decided to exclude this electrode from all further recordings and analyses. All EEG electrodes were nose-referenced and a forehead ground was used (Fpz). In general, the impedance of electrodes was kept below 15k Ω during the experiment. The setup time for the electrode configuration was 35 minutes on average.

All stimuli were presented on two 24’’ monitors (LG, Seoul, South Korea) at a refresh rate of 60 Hz and a resolution of 1920px x 1080px. Participants’ responses were collected using two RB-740 response pads (Cedrus Corporation, San Pedro, USA) with 6 buttons (number of 1–6) used on one pad and 2 buttons (“Truth” and “Lie”) used on the other pad. The facial expressions of the participants were recorded using an HD pro C920 webcam (Logitech, Lausanne, Switzerland). The experiment was implemented in Python with PsychoPy^18^. Data preprocessing was performed with MATLAB (The MathWorks, Natick, MA, USA) using EEGLAB^19^, further ERP analysis and statistical test was performed using the Berlin BCI toolbox^20^.

### Experimental Task

The experiment involved participants engaging in a card-based deception game, in which they played the role of either a “player” or an “observer” opposite a counterpart. Participants were paired based on similar risk-taking scores (obtained through the Balloon Analogue Risk Task or BART^17^), age, and gender.

**Fig. 1:**
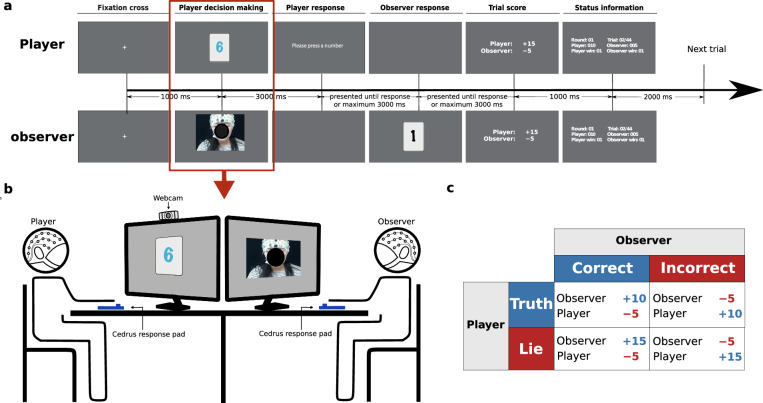
Experimental setup. (**a**) Trial structure of the experiment. The trial started with a 1-second fixation cross (1st figure), followed by the player’s 3-second decision-making phase where their face was shown via live camera stream to the observer while looking at the card (2nd figure). Next, the player was instructed to give the response with a maximum reaction time of 3 seconds (3rd figure). The player’s response was then shown to the observer, who was asked to decide between lie or truth within a maximum response period of 3 seconds (4th figure). The trial ended with a 1-second trial score and 2 seconds status information screen (5th and 6th figure). (**b**) Experimental setup with a schematic illustration of the situation in the decision-making phase of the player. The player and the observer sat face to face with monitors between them. Participants’ responses were collected using a response pad, and the player’s face was displayed in real-time to the observer via a webcam stream. (**c**) Payoff matrix of the game for the player and the observer.

During the game, the player and observer sat facing each other, separated by two monitors, as depicted in Fig. 1b. Each trial commenced with the player receiving a card displaying a number. The player’s task was to relay the number on the card to the observer. Relying on the player’s facial expressions and strategic considerations, the observer then determined whether the information provided was truthful or deceptive. Players were prompted to adapt their behavior based on the color of their assigned card, which indicated the response type. The color-response assignments were randomized for each participant. After completing one session, the roles of the player and observer were swapped for a second session.

The entire game consisted of 11 rounds, with each round comprising 44 trials. After each round, a 30-second break was given before the subsequent round began. Of the 44 trials in a round, 22 were spontaneous, 11 were instructed lies, and 11 were instructed truths, presented in a randomly shuffled order. The stimuli consisted of cards displaying numbers ranging from 1 to 6, each printed in one of three colors (black, purple, or blue), with the color assignment contingent on the instruction.

The game commenced with an explanation of the card color-response assignments to the player. As illustrated in Fig. 1a, every trial began with a 1-second fixation cross, succeeded by a 3-second display of a card at the screen’s center. The player was instructed to focus on the card and make a decision within the 3-second window, with their facial expressions simultaneously displayed to the observer in real time. Subsequently, the player was prompted to select the card number they wanted to convey to the observer. This selection could be influenced by the card color cue, requiring players to select a different number than the one displayed in the “instructed lie”(instL) condition, the same number in the “instructed truth” (instT) condition, or any number of their choice in the spontaneous condition (sponT, sponL). Upon the player’s response, a black card displaying the chosen number was shown to the observer, who then had 3 seconds to decide whether the information was a “lie” or the “truth”. After the observer’s response, feedback was provided on the screen, displaying scores or penalties for both participants based on the outcome of the trial. The scoring system, explained prior to the experiment, was designed to incentivize lying for the player and lie detection for the observer: the winner received +15 points and the loser −5 points if the player lied, or +10 points for the winner and −5 points for the loser if the player was truthful. Each trial concluded with a status screen showing the accumulated total score, number of trials won, rounds won, and game progress.

## Data Records

The complete set of raw behavioral data, raw EEG data, and preprocessed EEG data utilized in this study is readily accessible on Figshare for use^21^. EEG data is made available in the Brain Vision Recorder’s native format, encompassing “.eeg” for raw data, alongside “.vhdr” and “.vmrk” files for header and marker information, respectively. The naming convention for raw EEG files follows the output format of the BrainVision recorder, categorizing each participant by their role—either as a player or an observer—with filenames exemplified by Player_sub01.eeg and Observer_sub01.eeg.

Raw behavioral logs are presented as space-separated text documents (“.txt”), and accompanying event timestamp files (“.txt”), correlated with the EEG recordings, are prepared for each participant. These documents include information detailed in Tables 2 and 3. Behavioral and timestamp files are named to reflect the dyadic structure of the experimental sessions, indicating participant roles and numbers—for instance, Player_sub01_Observer_sub02_Behavioral.txt and Player_sub01_Observer_sub02_Timestamp.txt.

**Table 2:** Log information.

Category	Details
Demographic characteristics	Age
	Sex
Trial information	Card type (spon/inst)
	Card color
	Card number
	Trial number
	Round number
Response information	Player’s response (Truth, lie)
	Observer’s response (Truth, lie)
	player’s input number
	Player’s reaction time
	observer’s reaction time

**Table 3:** Timestamp information.

Category	Details
Stimulus	Round start
	Round end
	Fixation cross
	Card show (Spon/instL/instT)
	Player input start
	Observer input start
	Break start
	Break end
Response	Player input (truth/lie, number)
	Observer input (truth/lie)
Feedback	Trial status
	Trial result
	Round result
	Final game result

For the preprocessed EEG data and data prepared for 1D-CNN classification, filenames are systematically organized based on the type of stimulus onset, such as DecisionMaking (player decision making phase in Fig. 1a) or Feedback (Trial score in Fig. 1a), to facilitate targeted analysis. Preprocessed data files bear names that mirror individual session identifiers, like Player_sub01_Observer_sub02.mat. Conversely, Datasets for 1D-CNN classification are consolidated into single.mat files per session for efficient initial loading in Python, optimizing trial selection and data handling by mitigating the cumbersome loading of.mat file.

## Technical Validation

### Data preprocessing

**Fig. 2:**
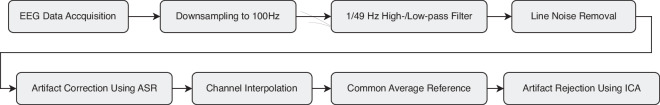
Flowchart of preprocessing steps.

As depicted in Fig. 2, the data underwent downsampling to 100 Hz, followed by the application of a 1/49 Hz high-/low-pass filter. Channel rejection was performed using EEGLab function clean_artifact() with channels whose line noise power was 4 standard deviations higher than their signals, and lower correlations than 0.85 with their reconstructed versions based on adjacent channels being rejected. With the same function, EEG data containing nonstationary high-amplitude bursts were removed using artifact subspace reconstruction (ASR)^22^, which is a principle component-based method. The ASR procedure was applied using a 500-ms sliding window and a lax (20 standard deviations) threshold that removes extreme mechanical artifacts while preserving brain signal components. This method has been shown to improve the quality of a subsequent Independent Component Analysis (ICA) decomposition^23,24^. Next, all removed channels were interpolated and EEG data were then re-referenced to a common average reference. ICA was performed using EEGLab function runamica15() in EEGLab and the independent components (ICs) were subsequently separated into several signal categories (e.g., brain, muscle, eye, etc.) by a trained classifier ICLabel^25^ using EEGLab function iclabel(). The ICs labeled as eye movements with probabilities higher than 0.7 were rejected.

EEG epochs were extracted for both the player and the observer. For the player, 3500 ms epochs were taken starting 500 ms before the onset of the stimulus presentation (Player decision making period in Fig. 1a). These epochs were grouped into four conditions: instructed truth (instT), instructed lie (instL), spontaneous truth (sponT), and spontaneous lie (sponL), with a baseline correction interval of 500 to 0 ms before stimulus onset. For the observer, 1200 ms epochs commenced 200 ms before the feedback (Trial score in Fig. 1a) onset. These epochs were categorized into two conditions: correct and incorrect, using a baseline correction interval of 200 to 0 ms. In both cases, subjects with artifact-free epochs were retained for analysis. However, one participant from each group had to be excluded due to faulty EEG equipment, leaving 23 participants in each category for ERP analysis.

### EEG analysis

#### ERP and statistical analysis

The ERP was calculated using a weighted average, as the spontaneous trials involving binary comparisons did not have balanced numbers of trials. Topographic maps of significant features for the four different deception conditions were calculated by point-biserial correlation coefficients^26^, measuring the association of the trial type label to the electrode-wise ERP data. Using Fisher’s transformation, correlations were transformed into unit variance *z*-scores for each subject, and grand average *z*-scores were obtained by weighted sums of individual *z*-scores over all subjects. In calculating grand-average statistics, inverse-variance weighting under a fixed-effects hierarchical model based on the sufficient statistics approach^27^ was used. *P*-values for the hypothesis of zero correlation in the grand average were computed using a two-sided *z*-test. All reported *p*-values were Bonferroni-corrected to account for multiple hypothesis testing.

#### Microstate

We also conducted a microstate analysis using the Randomization Graphical User Interface^28^. This approach uses a spatial K-means clustering approach to pinpoint functional microstates, characterized by quasi-stable scalp map topographies, by measuring global map dissimilarity^29^. Dominant topographies in the grand-mean stimulus/feedback-locked ERP map series for player and observer were identified. To ascertain the optimal cluster count, we assessed models of different cluster numbers on training data, comparing their mean correlation against the rest of the participants’ stimulus-locked ERPs. This process was executed 50 times, averaging results to accommodate inter-participant variance while minimizing intra-participant variance. the cluster count that best represented the group-averaged data were selected. A topographical fitting procedure was then employed to find the onset and offset of each microstate in the grand-mean stimulus-locked ERP up to the intersection point.

### Decoding analysis

**Fig. 3:**
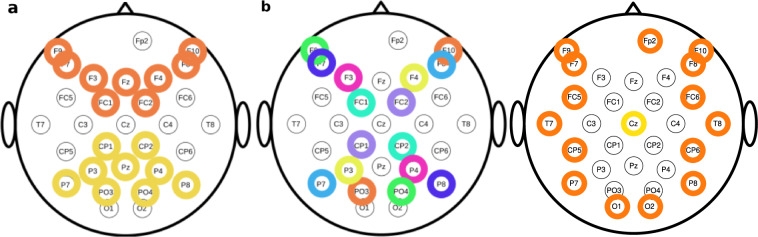
Selection of electrode pairs for 1D-CNN classification. (**a**) fronto-occipital symmetrical electrode pairs used for player. (**b**) x-pattern electrode pairs used for player. (**c**) circular design for observer (e.g. each pf the orange electrodes are paired to central yellow electrode).

**Fig. 4:**
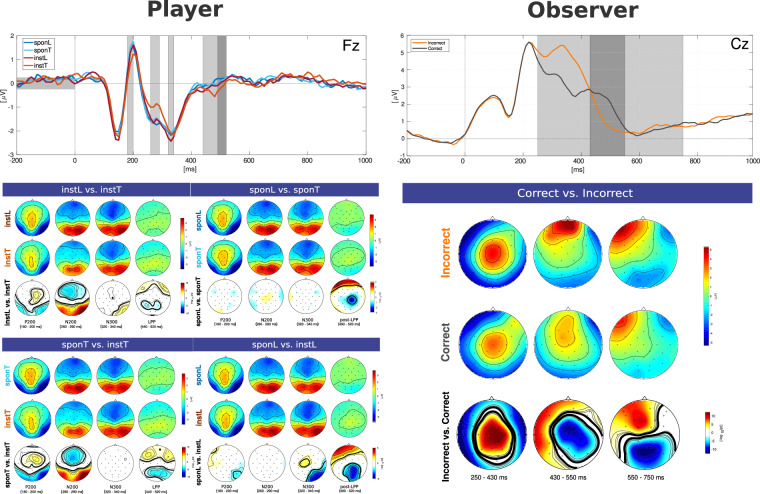
Event-related potential. Left) For the player, first row shows grand average ERP analysis of all conditions for electrodes Fz for Player and observer. Shaded areas indicate P200, N200, N300, LPP, and post-LPP (overlapped with LPP), respectively. Below shows the ERP scalp map (rows 1 and 2) and signed logarithm *p*-values (row 3) indicating the grand average statistical significance of difference for instL vs. instT (left top), sponL vs. instT (right top), sponT vs.instT (left bottom), and sponL vs. sponT (right bottom). Right**)** For the observer, top shows grand average ERP contrasing condition correct and incorrect. Shaded areas indicate P300, P400 and N500. bottom shows the ERP scalp map (rows 1 and 2) and statistical significance of difference for incorrect vs. correct. Bold contours indicate *p* < 0.05, where *p*-values are corrected for multiple comparisons.

**Fig. 5:**
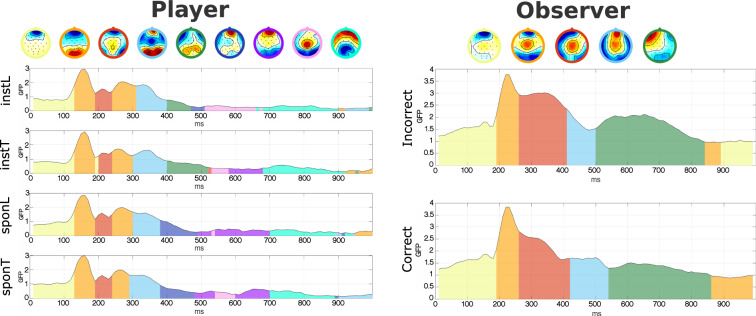
Microstate. Microstate clusters across time (ms) for all conditions plotted over global field power (GFP) for observer and player.

**Fig. 6:**
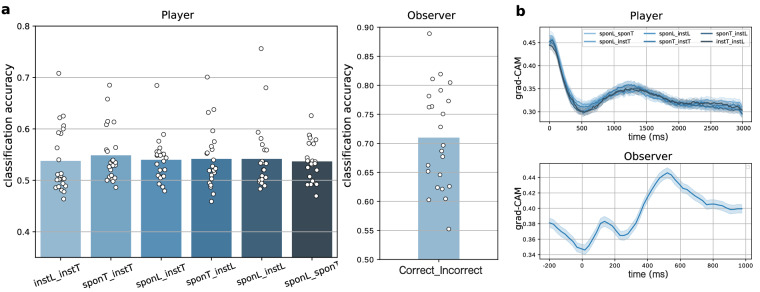
Single-Trial Clasiification result. (**a**) The mean cross-validated 1D-CNN classification accuracy for all six binary combinations of conditions (player) and correct vs. incorrect condition (observer) using 0 to 3000 ms post-stimulus data. Individual accuracy is indicated by white dots. (**b**) Feature importance calculated using Grad-CAM for all six binary combinations of conditions (player) and correct vs. incorrect condition (observer).

To assess single-trial decoding of the four different deception conditions, we used a 10-layer one-dimensional convolutional neural network (1D-CNN)^30^ in a stratified ten-fold cross-validation. This 1D-CNN efficiently extracts local features between adjacent elements in a feature vector^31^. This network, enhanced by an electrode selection strategy involving pairs of symmetrical electrodes in the region of interest^30,32^, outperformed prior CNN methods on motor imagery data. Based on significant ERP scalp patterns, we chose electrode pairs for training, as shown in Fig. 3, including frontal-occipital and X-pattern symmetrical electrodes relative to the transverse line through T7-C3-Cz-C4-T8. To verify if the 1D-CNN’s classification results exceeded chance levels, we conducted exact binomial tests within each participant for the six experimental conditions. The ratio of correct and incorrect predictions was compared to a null model with 0.5 accuracy (chance level). To understand the 1D-CNN classifier’s learned features, we adopted the Gradient-weighted Class Activation Mapping (Grad-CAM) approach^33^ to assess the impact of different time points. The feature importances from Grad-CAM were normalized in each training iteration within the ten-fold cross-validation Figs 4–6.

### Behavioral results

In spontaneous conditions, participants chose to either tell the truth or lie. We observed that the participants (N = 23) made significantly more truthful decisions (*mean* = 132, SD = ±20) than lying decisions (*mean* = 109, SD = ±20; *t*(22) = −3.809, *p* < 0.001). No significant correlation was found between the percentage of lies in the spontaneous condition and BART scores (*r* = −0.32, *p* = 0.13).

Regarding reaction times, previous research has shown that reaction data fits a convolution of normal and exponential distributions (Ex-Gaussian)^34^. A Box-Cox transformation^34^, with *λ* = 0.3, was therefore used to meet the normality assumption for parametric statistical tests. A 4-level (condition type: instL, instT, sponL, sponT) one-way repeated measure ANOVA found a significant main effect of condition type ($F(3,66)=4.8$, $p=0.0044$, ${\eta }_{p}^{2}=0.18$). Paired t-tests revealed participants responded significantly slower in the sponL condition ($G{M}_{untransformed}=552ms$) compared to all other conditions (instT: $G{M}_{untransformed}=513ms$, ${t}_{(sponL,instT)}(22)=2.95$, $p=0.0073$; instL: $G{M}_{untransformed}=521ms$, ${t}_{(sponL,instL)}(22)=2.97$, $p=0.0070$; sponT: $mean=502ms$, ${t}_{(sponL,sponT)}(22)=3.35$, $p=0.0029$). No significant differences were noted for other condition pairs.

### Benchmark EEG results

#### Event related potential

For the player, our ERP analysis revealed differences between the four experimental conditions in several components: P200, N200, N300, LPP, and post-LPP. When compared to the instT condition, other conditions elicited more pronounced P200 (170–200 ms) and N200 (240–290 ms) responses. P200 was related to emotional salience^35^, risky information^36^, or mismatch^37^, suggesting that participants required more attention in the instructed lie and spontaneous conditions. This increased attention could be attributed to the need for a choice in these conditions, whereas instT involved only a single button press. The N200, higher in instT, is associated with cognitive control and conflict monitoring, potentially due to greater cognitive control demands in deception^14,15,38^. Subsequent to the N200, an N300 difference was observed, only contrasting the instructed lie versus the spontaneous conditions, associated with more specific information in the presence of semantic incongruencies^39,40^. Our study suggests the N200 differentiates between cue-related default behavior and other behaviors, while the N300 distinguishes forced-choice deception from self-determined deception, with increased amplitude for forced-choice. The LPP, related to decision ambiguity^41,42^, was more pronounced in instL, sponT, and sponL, reflecting the graded ambiguity of choice in these conditions. Subsequent prefrontal post-LPP was higher in spontaneous conditions, likely due to the longer decision time in instructed lying and spontaneous decisions. A more pronounced post-LPP was observed for sponL, attributable to higher decision ambiguity. The lack of significant P200, N200, and N300 differences between sponL and sponT, despite visually identical cues, is consistent with previous studies^13–15^, suggesting that early LPP components are more perceptually driven. These results contribute to understanding the neural basis of deception, involving attentional control, cognitive control, semantic processing, and decision-making processes.

For the observer, post-feedback onset elicited the P300, P400, and a late negative potential N500. The P300, manifesting as a central positivity between 250–400 ms, is a widely studied feedback-related component linked to feedback type and valence. Consistent with prior research, P300 amplitudes showed heightened responses to negative feedback compared to positive ones^43,44^. The ensuing P400, identifiable by a positive deflection at frontal recording sites and a negative one at posterior sites, peaks around 400 ms post-feedback onset and was more pronounced in the loss condition. This P400 component has been linked to the processes of updating and memorizing information^45^. In the context of our study, this suggests observers were actively monitoring game scores to ensure a win in the game. Previous studies have indicated that the N500 is generally more pronounced for unpleasant stimuli and is believed to originate from the posterior cingulate cortex and visual association cortex^46,47^.

#### Microstate analysis

Our microstate analysis corroborated the main ERP patterns, showing consistently higher Global Field Power (GFP) for both players and observers. Specifically, players displayed unique clusters (5th and 6th) in the LPP interval (400–500 ms), which effectively distinguished between instructed and spontaneous conditions. In the instructed condition, we observed elevated posterior potentials, whereas the spontaneous condition yielded higher frontal potentials. For observers, a markedly higher GFP was linked to the P300 component in loss outcomes (p < 0.0005), further establishing P300 as the key component for differentiating between outcome types.

### Single-trial classification results

In our decoding analysis, we used 1D-CNN for single-trial classification to associate EEG features with behavioral performance. We achieved above-chance classification accuracy in all conditions for both players (55%) and observers(71%). Notably, Grad-CAM analysis highlighted that early ERP components (P200, N200, N300) were the key discriminative features for player classification. This confirms their ability to accurately distinguish between truths and lies in both instructed and spontaneous conditions. For observers, Grad-CAM indicated that later ERP components starting at 300 ms (P300, P400, N500) effectively classified correct and incorrect outcomes.

## Usage Notes

Our dataset, offering both player and observer perspectives in a competitive deception game, serves as a foundational resource for understanding cognitive functions during deceptive activities. The basic ERP analysis we performed has confirmed essential cognitive functions such as attention, cognitive control, decision ambiguity, and information processing for both players and observers, thereby setting the stage for more nuanced investigations. One immediate avenue for future research could involve exploring connectivity and inter-brain synchrony to investigate the dynamics within and between brains during deception. Our data could enable researchers to build more complex models of social interaction involving deception, potentially illuminating key neural pathways and mechanisms that govern truthful and deceptive behavior. As strategy plays a critical role in competitive games, future analyses could focus on how higher-order decision makings, like the dynamic adjustment of decisions based on previous outcomes, interact with basic cognitive functions^48,49^. This can extend to studies of game theory, risk taking, and decision-making in other contexts as well. Our robust single-trial classification results using 1D-CNN demonstrate the feasibility of employing deep learning for decoding players’ deceptions and observers’ feedback. Advanced deep learning models could further refine these techniques, contributing to not only more accurate, real-time decision-making and deception detection systems, but also for applications in cognitive rehabilitation.

## Data Availability

The EEG preprocessing, ERP analysis code and code used for classification is avaliable at https://github.com/yiyuchen-lab/DeceptionGame.

## References

[1] Lykken DT (1959). The GSR in the detection of guilt. Journal of Applied Psychology.

[2] Rosenfeld, J. P. P300 in detecting concealed information. *Memory detection: Theory and application of the Concealed Information Test* 63–89 (2011).

[3] Christ SE, Van Essen DC, Watson JM, Brubaker LE, McDermott KB (2009). The contributions of prefrontal cortex and executive control to deception: Evidence from activation likelihood estimate meta-analyses. Cerebral Cortex.

[4] Farah MJ, Hutchinson JB, Phelps EA, Wagner AD (2014). Functional MRI-based lie detection: Scientific and societal challenges. Nature Reviews Neuroscience.

[5] Lisofsky N, Kazzer P, Heekeren HR, Prehn K (2014). Investigating socio-cognitive processes in deception: A quantitative meta-analysis of neuroimaging studies. Neuropsychologia.

[6] Hsu C, Begliomini C, Dall’Acqua T, Ganis G (2019). The effect of mental countermeasures on neuroimaging-based concealed information tests. Human brain mapping.

[7] Lukács G (2016). The first independent study on the complex trial protocol version of the P300-based concealed information test: Corroboration of previous findings and highlights on vulnerabilities. International Journal of Psychophysiology.

[8] Kleinberg B, Verschuere B (2015). Memory detection 2.0: The first web-based memory detection test. PloS one.

[9] Peth J (2015). Memory detection using fMRI—does the encoding context matter?. NeuroImage.

[10] Winograd MR, Rosenfeld JP (2014). The impact of prior knowledge from participant instructions in a mock crime P300 concealed information test. International journal of psychophysiology.

[11] Sun D, Lee TM, Wang Z, Chan CC (2016). Unfolding the spatial and temporal neural processing of making dishonest choices. PloS one.

[12] Hu X, Pornpattananangkul N, Nusslock R (2015). Executive control-and reward-related neural processes associated with the opportunity to engage in voluntary dishonest moral decision making. Cognitive, Affective, & Behavioral Neuroscience.

[13] Panasiti MS (2014). The motor cost of telling lies: Electrocortical signatures and personality foundations of spontaneous deception. Social neuroscience.

[14] Sai L, Wu H, Hu X, Fu G (2018). Telling a truth to deceive: Examining executive control and reward-related processes underlying interpersonal deception. Brain and cognition.

[15] Carrión RE, Keenan JP, Sebanz N (2010). A truth that’s told with bad intent: An ERP study of deception. Cognition.

[16] Sacré P (2019). Risk-taking bias in human decision-making is encoded via a right–left brain push–pull system. Proceedings of the National Academy of Sciences.

[17] Chen, Y. & Wallraven, C. Pop or not? EEG correlates of risk-taking behavior in the balloon analogue risk task. In *2017 5th International Winter Conference on Brain-Computer Interface (BCI)*, 16–19 (IEEE, 2017).

[18] Peirce JW (2007). Psychopy—psychophysics software in python. Journal of neuroscience methods.

[19] Delorme A, Makeig S (2004). EEGLAB: an open source toolbox for analysis of single-trial EEG dynamics including independent component analysis. Journal of neuroscience methods.

[20] Blankertz B (2010). The berlin brain–computer interface: non-medical uses of BCI technology. Frontiers in neuroscience.

[21] Chen Y, Wallraven C, Fazli S (2024). figshare.

[22] Kothe, C. A. E. & Jung, T.-P. Artifact removal techniques with signal reconstruction (2016). US Patent App. 14/895,440.

[23] Chang C-Y, Hsu S-H, Pion-Tonachini L, Jung T-P (2019). Evaluation of artifact subspace reconstruction for automatic artifact components removal in multi-channel EEG recordings. IEEE Transactions on Biomedical Engineering.

[24] Artoni F (2017). Unidirectional brain to muscle connectivity reveals motor cortex control of leg muscles during stereotyped walking. Neuroimage.

[25] Pion-Tonachini L, Kreutz-Delgado K, Makeig S (2019). Iclabel: An automated electroencephalographic independent component classifier, dataset, and website. NeuroImage.

[26] Tate RF (1954). Correlation between a discrete and a continuous variable. point-biserial correlation. The Annals of mathematical statistics.

[27] Dowding I, Haufe S (2018). Powerful statistical inference for nested data using sufficient summary statistics. Frontiers in human neuroscience.

[28] Koenig T, Stein M, Grieder M, Kottlow M (2014). A tutorial on data-driven methods for statistically assessing ERP topographies. Brain topography.

[29] Koenig T, Melie-Garcia L (2010). A method to determine the presence of averaged event-related fields using randomization tests. Brain topography.

[30] Mattioli F, Porcaro C, Baldassarre G (2022). A 1D-CNN for high accuracy classification and transfer learning in motor imagery eeg-based brain-computer interface. Journal of Neural Engineering.

[31] Schirrmeister RT (2017). Deep learning with convolutional neural networks for EEG decoding and visualization. Human brain mapping.

[32] Lun X, Yu Z, Chen T, Wang F, Hou Y (2020). A simplified cnn classification method for MI-EEG via the electrode pairs signals. Frontiers in Human Neuroscience.

[33] Selvaraju, R. R. *et al*. Grad-CAM: Visual explanations from deep networks via gradient-based localization. In *Proceedings of the IEEE International Conference on Computer Vision*, 618–626 (2017).

[34] Marmolejo-Ramos F, Cousineau D, Benites L, Maehara R (2015). On the efficacy of procedures to normalize ex-gaussian distributions. Frontiers in psychology.

[35] Chou L-C, Pan Y-L, Lee C-l (2020). Emotion anticipation induces emotion effects in neutral words during sentence reading: Evidence from event-related potentials. Cognitive, Affective, & Behavioral Neuroscience.

[36] Ma Q, Jin J, Wang L (2010). The neural process of hazard perception and evaluation for warning signal words: evidence from event-related potentials. Neuroscience letters.

[37] Xiao F, Sun T, Qi S, Chen Q (2019). Common and distinct brain responses to detecting top-down and bottom-up conflicts underlying numerical inductive reasoning. Psychophysiology.

[38] Zeki S (2004). A cognitive neurobiological account of deception: evidence from functional neuroimaging. Philosophical Transactions of the Royal Society of London. Series B: Biological Sciences.

[39] Sitnikova T, Holcomb PJ, Kiyonaga KA, Kuperberg GR (2008). Two neurocognitive mechanisms of semantic integration during the comprehension of visual real-world events. Journal of cognitive neuroscience.

[40] Yum YN, Holcomb PJ, Grainger J (2011). Words and pictures: An electrophysiological investigation of domain specific processing in native chinese and english speakers. Neuropsychologia.

[41] Sun S (2017). Decision ambiguity is mediated by a late positive potential originating from cingulate cortex. NeuroImage.

[42] Sun, S., Yu, R. & Wang, S. A neural signature encoding decisions under perceptual ambiguity. *eneuro* **4** (2017). 10.1523/ENEURO.0235-17.2017 PMC5701297 29177189

[43] Frank MJ, Woroch BS, Curran T (2005). Error-related negativity predicts reinforcement learning and conflict biases. Neuron.

[44] Martnez-Selva JM, Muñoz MA, Sánchez-Navarro JP, Walteros C, Montoya P (2019). Time course of the neural activity related to behavioral decision-making as revealed by event-related potentials. Frontiers in Behavioral Neuroscience.

[45] Gui P (2018). Neural correlates of feedback processing in visuo-tactile crossmodal paired-associate learning. Frontiers in Human Neuroscience.

[46] Carretié L, Hinojosa JA, Albert J, Mercado F (2006). Neural response to sustained affective visual stimulation using an indirect task. Experimental Brain Research.

[47] De Pascalis V, Strelau J, Zawadzki B (1999). The effect of temperamental traits on event-related potentials, heart rate and reaction time. Personality and Individual Differences.

[48] Wang Y (2017). Social value orientation modulates the FRN and P300 in the chicken game. Biological psychology.

[49] Kirschner H, Fischer AG, Ullsperger M (2022). Feedback-related EEG dynamics separately reflect decision parameters, biases, and future choices. NeuroImage.

[50] Nieto N, Peterson V, Rufiner HL, Kamienkowski JE, Spies R (2022). Thinking out loud, an open-access EEG-based bci dataset for inner speech recognition. Scientific Data.

[51] Wilson H, Golbabaee M, Proulx MJ, Charles S, O’Neill E (2023). EEG-based BCI dataset of semantic concepts for imagination and perception tasks. Scientific Data.

[52] Hinss MF (2023). Open multi-session and multi-task EEG cognitive dataset for passive brain-computer interface applications. Scientific Data.

[53] Pavlov YG, Kasanov D, Kosachenko AI, Kotyusov AI, Busch NA (2022). Pupillometry and electroencephalography in the digit span task. Scientific data.

[54] Mheich A (2021). HD-EEG for tracking sub-second brain dynamics during cognitive tasks. Scientific Data.

[55] Chen Z (2023). Open access dataset integrating EEG and fNIRS during stroop tasks. Scientific Data.

[56] Chen Z, Ji X, Li T, Gao C, Liu S (2023). Lateralization difference in functional activity during stroop tasks: a functional near-infrared spectroscopy and eeg simultaneous study. Frontiers in Psychiatry.

[57] Chen K (2022). A resource for assessing dynamic binary choices in the adult brain using eeg and mouse-tracking. Scientific data.

[58] Cao Z, Chuang C-H, King J-K, Lin C-T (2019). Multi-channel EEG recordings during a sustained-attention driving task. Scientific data.

[59] Dzianok P, Antonova I, Wojciechowski J, Dreszer J, Kublik E (2022). The nencki-symfonia electroencephalography/event-related potential dataset: Multiple cognitive tasks and resting-state data collected in a sample of healthy adults. GigaScience.

